# Trained Immunity Contribution to Autoimmune and Inflammatory Disorders

**DOI:** 10.3389/fimmu.2022.868343

**Published:** 2022-04-08

**Authors:** Samanta C. Funes, Mariana Rios, Ayleen Fernández-Fierro, María S. Di Genaro, Alexis M. Kalergis

**Affiliations:** ^1^Instituto Multidisciplinario de Investigaciones Biológicas-San Luis (IMIBIO-SL), Consejo Nacional de Investigaciones Científicas y Técnicas (CONICET), Universidad Nacional de San Luis (UNSL), San Luis, Argentina; ^2^Millennium Institute on Immunology and Immunotherapy, Departamento de Genética Molecular y Microbiología, Facultad de Ciencias Biológicas, Pontificia Universidad Católica de Chile, Santiago, Chile; ^3^Departamento de Endocrinología, Escuela de Medicina, Facultad de Medicina, Pontificia Universidad Católica de Chile, Santiago, Chile

**Keywords:** trained immunity, autoimmunity, autoinflammation, vaccines, trained immune cells, BCG (Bacille Calmette-Guérin)

## Abstract

A dysregulated immune response toward self-antigens characterizes autoimmune and autoinflammatory (AIF) disorders. Autoantibodies or autoreactive T cells contribute to autoimmune diseases, while autoinflammation results from a hyper-functional innate immune system. Aside from their differences, many studies suggest that monocytes and macrophages (Mo/Ma) significantly contribute to the development of both types of disease. Mo/Ma are innate immune cells that promote an immune-modulatory, pro-inflammatory, or repair response depending on the microenvironment. However, understanding the contribution of these cells to different immune disorders has been difficult due to their high functional and phenotypic plasticity. Several factors can influence the function of Mo/Ma under the landscape of autoimmune/autoinflammatory diseases, such as genetic predisposition, epigenetic changes, or infections. For instance, some vaccines and microorganisms can induce epigenetic changes in Mo/Ma, modifying their functional responses. This phenomenon is known as trained immunity. Trained immunity can be mediated by Mo/Ma and NK cells independently of T and B cell function. It is defined as the altered innate immune response to the same or different microorganisms during a second encounter. The improvement in cell function is related to epigenetic and metabolic changes that modify gene expression. Although the benefits of immune training have been highlighted in a vaccination context, the effects of this type of immune response on autoimmunity and chronic inflammation still remain controversial. Induction of trained immunity reprograms cellular metabolism in hematopoietic stem cells (HSCs), transmitting a memory-like phenotype to the cells. Thus, trained Mo/Ma derived from HSCs typically present a metabolic shift toward glycolysis, which leads to the modification of the chromatin architecture. During trained immunity, the epigenetic changes facilitate the specific gene expression after secondary challenge with other stimuli. Consequently, the enhanced pro-inflammatory response could contribute to developing or maintaining autoimmune/autoinflammatory diseases. However, the prediction of the outcome is not simple, and other studies propose that trained immunity can induce a beneficial response both in AIF and autoimmune conditions by inducing anti-inflammatory responses. This article describes the metabolic and epigenetic mechanisms involved in trained immunity that affect Mo/Ma, contraposing the controversial evidence on how it may impact autoimmune/autoinflammation conditions.

## Introduction

Classically the immune response in vertebrates has been classified as innate and adaptive. The latter requires the presence of B and T lymphocytes that, when faced with a pathogen, mount a specific response and establish a memory. Although this process requires time (days), a faster and more effective specific response takes place after subsequent antigen encounters. On the other hand, innate responses have historically been characterized by constitutive systems, such as complement and phagocyte activity, which are non-specific, run rapidly (hours), and do not establish a memory (1). However, a large body of evidence supports that exposure to pathogens can induce a memory-like response in the innate system (2, 3), as we will discuss below in this review.

**Figure 1:**
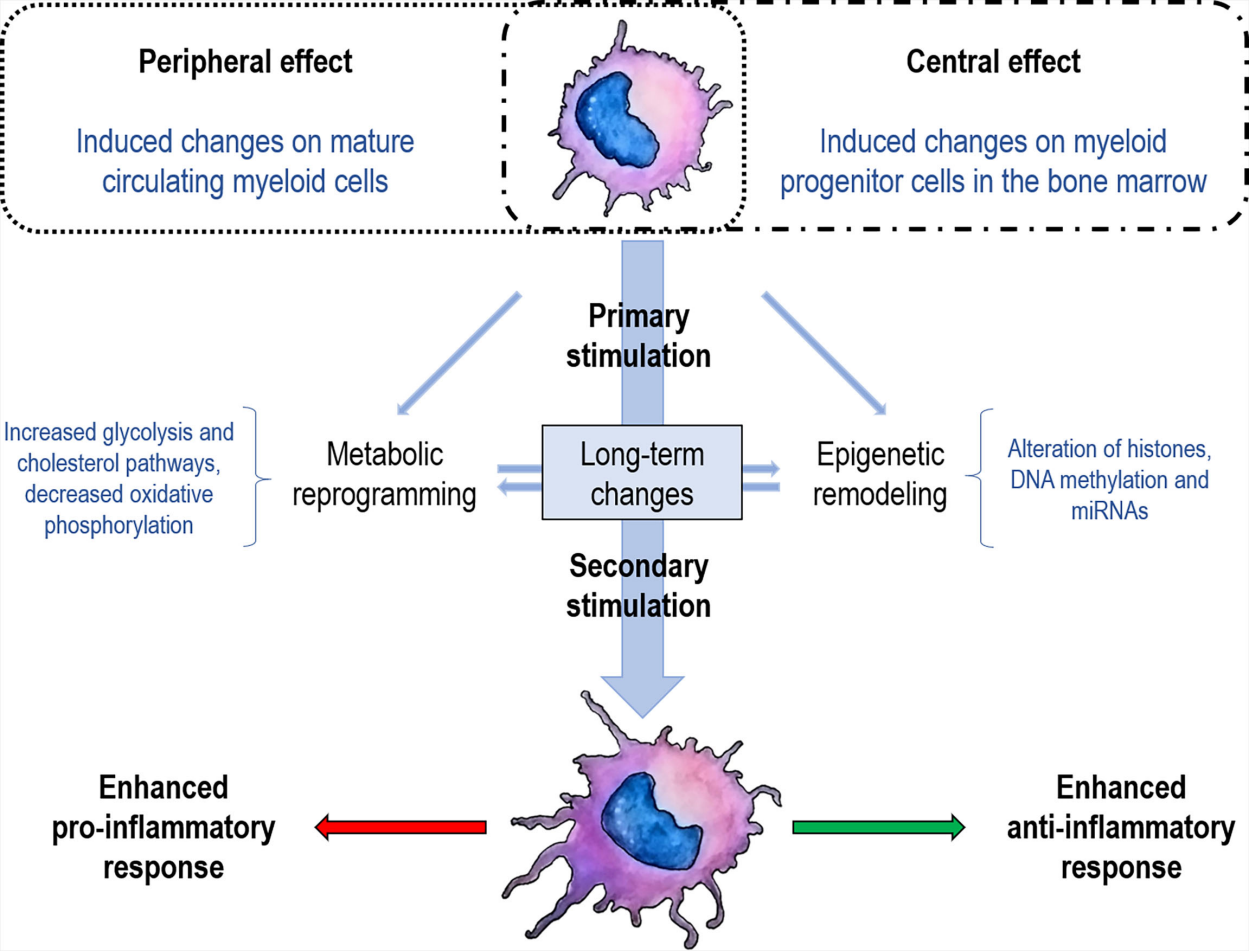
Schematization of trained immunity concepts. The first encounter with a specific stimulus (vaccine, glucan, pathogen) determines metabolic changes and establishes an epigenetic scar either in mature cells (peripheral training) or in stem cells (central training). These marks enable trained immunity to a strengthened response when facing a second stimulus, either to an increase of the pro-inflammatory or anti-inflammatory response. Although the pro-inflammatory response has been the most documented for trained immunity, the anti-inflammatory response has recently been described.

Trained immunity was first proposed in 2011 as the enhanced innate immune response during a second encounter with the same or different microorganisms (cross-protection) (4). It should be noted that this innate memory differs from adaptive memory since it is not antigen-specific and is based on the strengthening of the innate response to the subsequent encounter with pathogens. This type of immune response cannot be classified as either innate (only observed in the second encounter) or as adaptive (no specific memory itself), so it is defined as another mechanism occurring after a second encounter (4). Trained immunity is mainly mediated by Mo/Ma and NK cells independently of T and B cells. The improvement in cell function in these cell populations is related to epigenetic and metabolic changes that modify gene expression and the phenotype of these cells (**Figure 1**). Although the benefits of immune training have been extensively highlighted in a vaccination context, its effects on autoimmunity and chronic inflammation are still controversial.

## Brief Description of Training Mechanisms

A type of innate memory against previous inflammatory events has been described in plants (5) and various invertebrates as an adaptation to the lack of an adaptive immune system (6). In the case of vertebrates, the stimulation of innate cells through different pattern recognition receptors (PRRs), such as Toll-like receptors (TLRs), nucleotide-binding oligomerization domain-like receptors or NOD-like receptors (NLRs), or C-type lectin receptors (CLRs) promotes long-term modifications that modulate cell metabolism and epigenetic reprogramming (7, 8). PRRs interact with pathogen-associated molecular patterns (PAMPs) and damage/danger-associated molecular patterns (DAMPs). However, the specificity of these receptors can also recognize non-harmful elements generating sterile inflammation (9). For example, macrophages express dectin-1 and TLR-4, recognizing the DAMPs vimentin and high-mobility group box protein-1 (HMGB-1), respectively. These molecules are secreted under injury situations and can train macrophages, making them prone to produce IL-6 and TNF under a second stimulus (10). Therefore, a strict balance between pro- and anti-inflammatory responses is required to avoid chronic inflammation or immune paralysis (11).

Metabolically, cells in a quiescent state have low biosynthetic demand and mainly metabolize glucose *via* glycolysis coupled with oxidative phosphorylation (12). Thus, circulating monocytes in the resting state mostly use the Krebs cycle to synthesize essential molecules or oxidative phosphorylation (13). However, once the cells are activated, they produce biosynthetic precursors by increasing glucose consumption through aerobic glycolysis and oxidative phosphorylation (14). Thus, it has been observed that β-glucans (cell wall components of fungi that are prototype agonists that induce trained immunity) produce a shift in cellular metabolism from oxidative phosphorylation to aerobic glycolysis in monocytes (15). This increased metabolic activity raises the synthesis of metabolites that modulate long-term innate immunity (15, 16).

Metabolic processes, such as glycolysis and fatty acid metabolism can influence immune cell function rather than simply generating energy or modulating general biosynthesis (17). In fact, metabolic reprogramming joins other key immunoregulatory events that influence the immune response (18). Metabolic flexibility in cells is essential to respond to critical changes in the environment and functional demands. In other words, cells can reprogram their metabolism due not only to changes in the availability of nutrients but also in response to the signaling by PRRs and other receptors (cytokine and antigen receptors) (18). Thus, the shift toward aerobics glycolysis and fatty acid synthesis away from the Krebs cycle and fatty acid oxidation is a feature of activated macrophages and DCs (19). Thus, in various immune cells, the increment in glycolysis leads to immune activation contrary to the induction of fatty acid oxidation, oxidative phosphorylation (OXPHOS), and lipid uptake that contribute to immune suppression (20).

Moreover, macrophages are highly plastic cells that can adopt a pro-inflammatory (classical or M1) or anti-inflammatory (alternative or M2) profile, and in each case, their metabolic commitment is adapted accordingly (21). For example, in M1 macrophages aerobic glycolysis predominates while the M2 macrophages engage with OXPHOS and the Krebs cycle (22). As macrophages, DCs undergo cellular changes (morphology, synthesized cytokines, antigenic presentation, increased glycolysis) that define their activated state after stimulation by PRRs (23). Furthermore, the formation of neutrophil extracellular traps (NETs) by neutrophils is dependent on glycolysis, and their activation with PMA increases glucose uptake (24). Similarly, NK cells are activated in the periphery, increasing glucose uptake, glycolysis, and lipid synthesis (25).

On the other hand, despite most of the metabolic studies focusing on glucose pathway shifts, there have also been reports of increased cholesterol synthesis in trained immunity. Consistently with this notion, it was observed that the induction of this pathway is crucial for the establishment of innate memory (26). Furthermore, inhibition of cholesterol synthesis pathways block the trained immunity seen from β-glucan exposure (26), and a deficiency in mevalonate kinase (MVK) associates with a constitutive phenotype of trained immunity and greater susceptibility to sterile inflammation (26).

**Figure 2:**
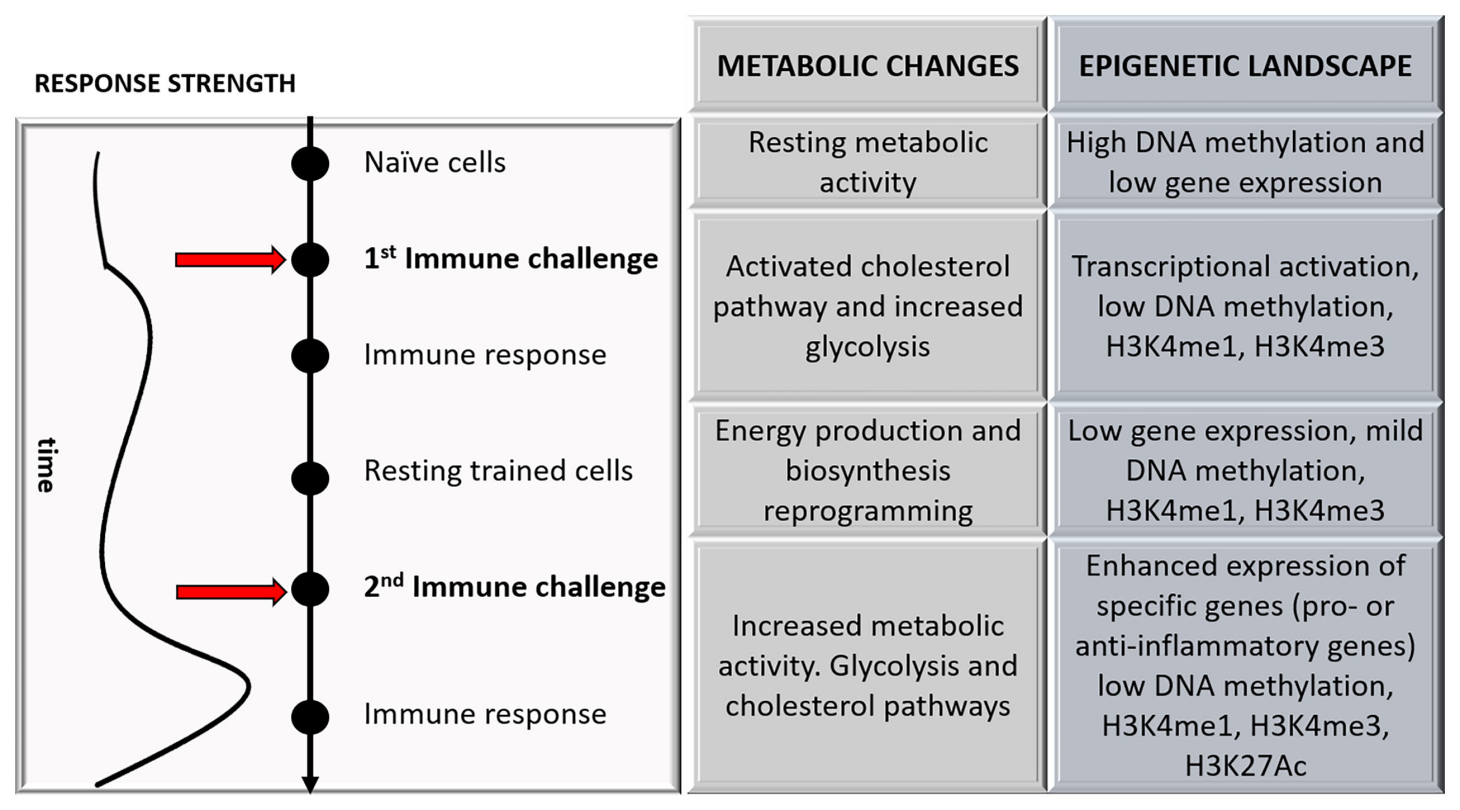
A schematic representation of the changes that occurred during trained immunity over time is shown, focusing on the major metabolic and epigenetic changes.

The above-mentioned metabolic changes are not isolated events within the cellular networks because these changes are closely related to epigenetic alterations capable of regulating innate immune memory (**Figure 2**). This is partly because many epigenetics events are closely associated with metabolic pathways by producing substrates and cofactors required for enzymatic activities (27). Accordingly, it has been observed that epigenetic modifications depend on cellular metabolism changes (16), and these modifications are blocked when metabolic changes are avoided (15, 28). Along these lines, the activation of the cell produces variations in the levels of various intracellular metabolites that lead to changes in the activity of specific enzymes responsible for modifying or reading the modifications in histones or DNA (29). For example, although the mechanisms are not yet fully understood, it is thought that there is a relationship between acetyl-CoA levels and histone acetylation (30). Besides, the accumulation of metabolic intermediates of the Krebs cycle, such as fumarate, inhibits demethylases increasing epigenetic changes in histones and trained immunity in monocytes (31).

On the contrary, the increase in itaconate, a product of the Krebs cycle, reduces epigenetic marks leading to immune tolerance after stimulation with PAMPs (32). Epigenetic regulation refers to phenotype changes without genotype alterations and includes both transient and stable structural alterations of the chromatin that impact gene expression (33). The mechanisms include various post-transcriptional modifications on histones (methylation, acetylation, among others), DNA chemical modifications, and regulation of non-coding RNAs (34). During primary stimulation of innate cells, active gene transcription is made possible by chromatin decondensation that facilitates access of the transcription machinery to DNA. The challenge of monocytes with stimuli such as β-glucans produces a long-lasting enrichment with marks such as the methylation of lysine (K) 4 or the K27 acetylation on histone H3 (H3K4me and H3K27ac, respectively) in the promoters of pro-inflammatory genes, increasing their expression (**Figure 2**) (35).

The effect induced on myeloid cells depends on the nature of the stimulus (the receptor involved) and on the concentration at which the exposure occurs. In this way, the same component can induce an attenuated or strengthened response when used in different concentrations (31). The major TLRs and NLR microbial ligands have been evaluated and their ability to attenuate or enhance the immune response in monocytes to a second encounter (36). These data show that muramyl dipeptide (MDP) and flagellin can induce trained immunity, and the latter is of particular interest because it has been assigned relevance to the pathogenesis of inflammatory bowel diseases (36). Thus, repeated exposure to LPS can induce selective and transient alterations in histones that repress the expression of pro-inflammatory factors in murine macrophages, favoring tolerance and reducing tissue damage by excessive inflammation (37).

On the other hand, exposure to *Candida albicans* or β-glucans induces stable epigenetic changes based on H3K4me, producing expression of inducible genes (35). Thus, β-glucan can at least partially reverse LPS-induced tolerance in Mo/Ma through changes in histones and reactivation of non-responding genes (38). Besides, stimulation with β-glucans in human monocytes produces both H3K4me3 and H3K27ac after seven days, and these changes were associated with induction of the glycolysis pathway (15).

Considering that trained immune response has been described several months after the first encounter (39), and due to the short life span of circulating Mo/Ma and NK cells, the question initially arose about how long-term reprogramming is established in these cells (4). In turn, this programming can be carried out at various levels of cellular function and locations, as will be mentioned in the following sections.

Importantly, trained immunity can be established peripherally in circulating mature cells or centrally in bone marrow progenitor cells, thus maintaining immune training for long periods (**Figure 1**). Different stimuli were shown to induce systemic changes, affecting hematopoiesis and reprogramming progenitor cells in the bone marrow (40, 41). Induction of trained immunity reprograms cellular metabolism in hematopoietic stem cells (HSCs), transmitting a memory-like phenotype to the cells (40, 42, 43). Thus, trained Mo/Ma derived from HSCs typically present a metabolic shift toward glycolysis, which leads to the modification of the chromatin architecture by methylases and acetylases (40). Stem cells express receptors for many inflammatory elements, allowing them to sense and adjust to changes in the environment (44). For example, acute stimulation with LPS induces persistent alterations in specific myeloid lineage enhancers, improving innate immunity against *P. aeruginosa* by a C/EBPβ dependent mechanism (45).

Similar to some infections, vaccines have also been reported to induce trained immunity, conferring non-specific protective effects against other non-related infections. For example, using the Bacillus Calmette-Guérin (BCG) vaccine, metabolic and epigenetic alterations were observed in monocytes both *in vivo* and *in vitro* (16, 46, 47). The result of the exposure of monocytes to the BCG vaccine or β-glucan is an increased cross-response (higher cytokine production) to subsequent exposure to another unrelated pathogen seven days later (35, 48, 49). Below we will detail the most important elements that induce trained immunity and the mechanisms that have been described for each of them.

### Vaccines

Vaccines have been developed to induce a specific immune response against a wide variety of pathogens for which they were designed. However, some vaccines can also protect against other pathogens with no specific vaccine by eliciting immune responses related to the concept of trained immunity (46, 47, 50). Furthermore, trained innate cells can boost vaccine strategies by increasing antigen uptake, presentation, migration, and cytokine production (51).

BCG, the vaccine for tuberculosis, has reduced mortality by decreasing morbidities other than tuberculosis in Africa (52). Interestingly, in the current pandemic against SARS-CoV-2, those countries where BCG vaccination is given at birth, it has been shown to have fewer COVID-19-related deaths and a lower contagion rate (46, 47, 53). Accordingly, BCG-vaccinated mice also increase their immune response against *C. albicans* or *Schistosoma mansoni*, at least in part through a T-independent mechanism (54). Moreover, BCG can improve vaccine performance against viral infections, such as influenza and hepatitis B, by enhancing cytokine production in humans and mice (55). It was shown that three months after BCG vaccination, the production of pro-inflammatory cytokines increased following *ex vivo* stimulation of NK cells with mycobacteria and other unrelated pathogens (48). Furthermore, in response to unrelated bacterial and fungal pathogens, through epigenetic reprogramming of innate immune cells, BCG increased not only the production of IFN-γ but also augmented the release of monocyte-derived cytokines, such as TNF and IL-1β (56). On the other hand, the induction of glycolysis and glutamine metabolism, regulated by epigenetic mechanisms at the chromatin organization level, has been demonstrated to be essential underlying BCG-induced trained immunity in monocytes both in an *in vitro* model and after vaccination of mice and humans (16).

The occurrence of trained immunity has also been observed in live-attenuated vaccines other than BCG, such as vaccines against smallpox (vaccinia virus), measles, polio (live oral vaccine), yellow fever, and the new live-attenuated *M. tuberculosis* candidate vaccine *MTB*VAC (57–62).

**Figure 3:**
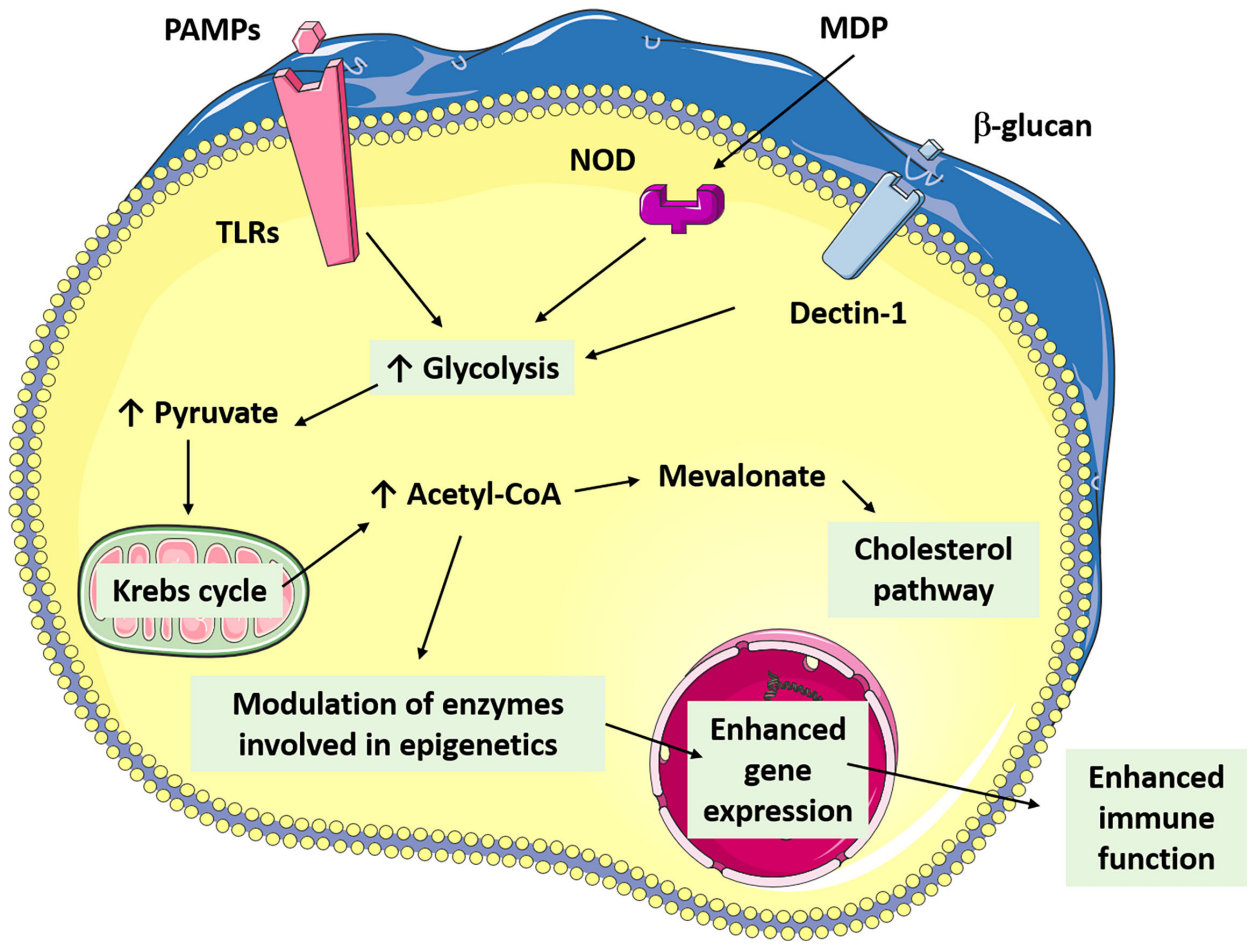
The figure shows in a simplified way the connection of the main metabolic pathways involved in the establishment of trained immunity through the commitment to some PAMPs (β-glucans, BCG, LPS).

The stimulus involved in the induction of trained immunity by vaccines is unclear. However, it is assumed that the immunogen from the vaccine can reach the bone marrow, where the hematopoietic stem and progenitor cells are stimulated, detecting PAMP, or could be indirectly stimulated by detecting systemic inflammatory signals like growth factors and cytokines such as GM-CSG, M-CSG, G-CSF, IL-1β, IL-6 (51). For instance, the bioactive peptidoglycan motif common to all bacterial vaccines is MDP (63), which activates innate cells through PRRs and leads to inflammatory cytokine release. Besides, BCG employs the mechanistic Target of Rapamycin (mTOR) pathway to activate specific downstream metabolic reprogramming and epigenetic changes (16). To date, the only intracellular PRR identified to be involved in the induction of trained immunity is NOD2/Rip2 in response to BCG (**Figure 3**). Moreover, activation of NOD2 (63) stimulates epigenetic changes in macrophages and induces trained immunity (56).

### Products Derived From Lipid Metabolism

The endotoxin LPS is the main outer membrane component of Gram-negative bacteria (64) and can regulate innate immune memory by promoting either inflammation or tolerance depending on acute or chronic stimulation and doses (37, 65). For instance, repeated low doses of LPS can increase the inflammatory response in mice after a stroke through changes in H3K4me1 in microglia (65). Moreover, acute stimulation with LPS induced persistent alterations in specific myeloid lineage, improving innate inflammatory immunity against *Pseudomonas aeruginosa* by a C/EBPβ dependent mechanism (45).

Other studies have shown that low doses of LPS reduced the expression of costimulatory molecules and increased expression of iCOS-ligand and DC-SIGN, promoting a mixed M1/M2 phenotype (66). Macrophages with a tolerogenic profile alleviated fibrosis and inflammation in a mouse model of systemic sclerosis (SSc), even by adoptive transfer (66). The gene response after LPS treatment is dynamically regulated to confer the tolerance phenotype (67). Thus, acetylation and methylation of histones are reconfigured to diminish transcription of pro-inflammatory cytokines, lipid metabolism, and phagocytic pathways (38, 67).

On the other hand, non-microbial molecules have been studied as trained immunity inducers, such as endogenous atherogenic particles (68–70). Compared to PAMPs, DAMPs have been much less studied as inducers of trained immunity. DAMPs are host-derived molecules capable of inducing an innate immune response. For example, oxidized low-density lipoprotein (oxLDL) is recognized by receptors as lectin-like oxidized low-density lipoprotein receptor-1 (LOX-1), CD36, and scavenger receptor class B type I (SR-BI) and, after a brief exposure, enhances the long-term pro-inflammatory response in Mo/Ma (69). Thus, oxLDL-trained macrophages present an epigenetic reprogramming associated with mTOR signaling showing high histone methylation and a pro-inflammatory profile (68). In addition, large containers of mitochondria have been found after exposure to ox-LDL, which correlates with an increased oxidative phosphorylation activity (69).

Hyperlipidemia has recently been studied as a factor to modulate or induce the inflammatory trained immune cell response (71), but the specific mechanisms regarding this matter remain unknown. A positive correlation between lipid concentration and the induction of metabolic genes has been observed, contributing significantly to trained immunity (70). Hyperlipidemia is linked with the increase of lysoPC and oxLDL as a stimulus for trained immunity (70); however, it has also been linked with increased aldosterone and liver X Receptor (LXR) activation, both proposed as part of a mechanism promoting trained immunity (72, 73).

### β-Glucan

The β-glucans are iconic inductors of trained immunity described throughout the literature (35, 74–77). β-glucans are glucose polymers found in the cell wall of fungi, rich in D-glucose units with β-1,3 links and β-1,6 branching, and recognized as PAMPs by dectin-1 in macrophages (**Figure 3**) (78). It has been reported that β-glucans from different sources (algae, yeast, bacteria, oat, and mushroom) can induce a strengthened response *in vitro* in peripheral blood mononuclear cells (PBMCs) (74). In murine models lacking functional T and B lymphocytes, trained immunity can be observed using *C. albicans* and fungal cell wall β-glucan. In that model, β-glucans induce functional reprogramming of monocytes, leading to augmented cytokine production and lower mortality under reinfection (35). Accordingly, the β-glucan treatment produces subsequent protection against *Staphylococcus aureus* infections in mice (75). Similarly, macrophages trained with β-glucan can protect mice against a *P. aeruginosa* infection (76).

The early inflammatory response induces epigenetic and metabolic changes, but interestingly, in this report, the induced immunity was independent of dectin-1 and TLR2 (76). Notably, β-glucan-mediated induction of training in macrophages requires cAMP production and activation of the mTOR-HIF1α pathway and aerobic glycolysis, similar to BCG trained immunity mechanisms (15, 79). Consistently, when glycolysis and glutaminolysis were inhibited, a reduction in histone marks was observed at the promoters of IL-6 and TNF (26). Besides, DCs also have shown enhanced response after fungal exposure. Mice exposed to *Cryptococcus neoformans* have DCs with strong IFN-γ production on a challenge and epigenetic changes (77).

β-Glucan-trained human monocytes undergo chromatin restructuring, identified by increased levels of H3K4me3, H3K27ac, and H3K4me1, as well as DNA demethylation, and increased accessibility of specific transcription factors at gene promoters corresponding to inflammation mediators (38, 79). Importantly, it has been observed that β-glucans also influence the myeloid progenitors of the bone marrow by producing epigenetic remodeling (80). Moreover, β-glucan promotes the expansion of myeloid-biased CD41+ HSCs in mice (41) and induces changes in HSCs in an IL-1β dependent manner (41).

## Beneficial and Detrimental Effects of the Induction of Trained Immunity for Autoimmune Disorders

Based on the above, trained immunity could play a pivotal role in defense against pathogens and even cancer cells, which is why it has been proposed as possible new immunotherapy (81). However, it is still debated whether this enhanced phenotype of innate cells could also contribute to establishing or maintaining chronic inflammatory conditions (82). Although the contribution of the genetic profile in the development of autoimmunity and the critical role of the adaptive immune system is well known, the innate response also plays essential functions in these conditions (83, 84). Autoimmune diseases are pathologies in which the immune response is unbalanced, characterized by autoreactive T and B cells, and complex pathogenesis with multifactorial etiology. Thus, immune cells recognize and attack the healthy tissues in a systemic or organ-specific manner, generating even more chronic inflammation (85). Along these lines, alterations in innate response characterized by a pro-inflammatory profile have also been described in innate cells from patients with autoimmune diseases (83, 86).

Two main scenarios can be identified with regard to trained immunity and autoimmune diseases. First, multiple factors, such as epigenetic alterations could be related to the observed inflammatory profile in autoimmune diseases (33, 87) and suggest a “training” state in the innate system under established autoimmunity. These findings have led to proposing new therapeutic strategies based on reversing metabolic or epigenetic changes to reduce or reverse the enhanced inflammatory state of the immune system (88). Second, the promotion of trained immunity could be harmful to individuals prone to developing autoimmune diseases. Therefore, a trained immunity signature in lupus mice and patients has been suggested by the reported reprogramming of HSCs towards the myeloid lineage that could contribute to exacerbated immune responses and flares in systemic lupus erythematosus (SLE) patients (87). Thus, SLE inflammatory milieu could promote immune training memory on bone marrow progenitor cells, similar to the observed β-glucan signature of HSCs after training (41). Besides, exposure to *Candida* β-glucans in two lupus-prone mouse models (*FcGRIIB*^-/-^ and pristane) increased the production of NETs and exacerbated disease activity (**Table 1**) (89). Accordingly, the administration of β-glucans in a lupus mouse model (NZBxNZW F1) has been reported to produce a more aggressive disease (90) and raises the re-evaluation of these components as immunomodulatory therapy in human lupus patients.

**Table 1:** Selection of reports that describe the effects of stimuli that produce trained immunity in patients or *in vivo* models of autoimmunity and the key molecules involved. The administration route is described as well.

Condition	Model	Stimuli	Route of administration	Immune training effect and key molecules involved	References
SLE	FcGRIIB-/- and pristane female mice	*Candida albicans*	Oral	Increased production of NETs and exacerbated disease activity. Induction of prominent NETs formation by Syk and NFκB expression in neutrophilic.	Saithong et al., (89)
	Female NZBxNZW F1 mice	β-glucans from *Saccharomyces cerevisiae*	Oral	More aggressive disease. The involvement of TLRs is suggested.	Fagone et al., (90)
RA	Female SKG mice	β-glucans (Zymosan)	Intraperitoneal	Trigger severe chronic arthritis with a higher incidence. β-glucans stimulate BM-DCs to mature and produce pro-inflammatory cytokines in a Dectin-1- but not TLRs dependent way.	Yoshitomi et al., (91)
	Male CIA model in DBA/1, DBA/2, BALB/c, C57BL/6, C3H/HeN and C57BL/10 mice	Particles containing β-glucan prepared from *Candida albicans* by oxidation	Subcutaneous or intraperitoneal	Exacerbate autoimmune arthritis. Genetic background (MHC and complement system) influences the ability of β-glucans as adjuvants.	Hida et al., (92)
	Male CIA model in DBA/1J mice	β-glucans derived from *Aureobasidium pullulans*	Intradermal	Inhibition of histopathological changes in CIA. Molecular mechanisms are unknown.	Kim et al., (93)
T1D	Female NOD/Mrk/TacfBR mice and new-onset diabetic patients	CFA or BCG	Intracutaneous	Inhibited the development of clinical diabetes in mice and clinical remission was observed in BCG-treated patients. Molecular mechanisms are not mentioned.	Shehadeh et al., (94)
	Healthy and diabetic subjects	BCG	Intradermal	Insulin-autoreactive T cell expansion and transient restoration of C-peptide. Mechanism related to TNF-induced death of insulin-autoreactive T cells.	Faustman et al., (95)
	Female NOD mice	CFA	Intradermal	CFA induces TNF-α production, a consequent elimination of TNF-α–sensitive cells and reverses the early stages of disease.	Ryu et al., (96)
SSc	Female HOCl-induced SSc mice	LPS and BCG	Intraperitoneal	Low-dose LPS alleviates fibrosis and inflammation, but BCG-training exacerbates disease. BCG-macrophages enhance the expression of pattern recognition receptors (TLR4, CD206, and CD14), chemokine receptors (CCR2 and CXCR4), costimulatory and/or signalling molecules (CD43, CD14, CD40, CD80, CD68, and Ly6C) and pro-inflammatory cytokines release (IL-6, TNF, and IL-1β). LPS^low^-macrophages express less costimulatory receptors and pro-inflammatory cytokines but upregulate IL-10, iCOS-ligand and DC-SIGN.	Jeljeli et al., (97)
MS	EAE in C57BL/6 mice	*Fasciola hepatica* total extract	Subcutaneous or intraperitoneal	FHTE increased the expression of *arg1*, *retlna*, *chi3l3*, CD206 and PD-L2 and the secretion of IL-1RA and IL-10 by macrophages while inhibiting TNF and IL-12p40 production in response to a TLRs restimulation. Besides, FHTE trained macrophages suppressed IL-17 production by T cells.	Quinn et al., (98)
	EAE in female C57BL/6 mice	*F. hepatica* excretory-secretory products (FHES)	Subcutaneous	Delay in the induction of murine EAE. FHES activates metabolic pathways (including mTOR) in HSCs, and the BMDM from FHES-treated mice reduces the production of pro-inflammatory cytokines and MHC-II expression but enhances IL-1RA. Besides had reduced costimulatory molecules expression and enhanced TGF-b, IL-10, IL-1R, and IL-6 production.	Cunningham et al., (99)
	EAE in female CD45.2 C57Bl/6J mice	CpG	Intravenous	Protection against EAE development by migration of pre-pDCs to the spine. BM cells stimulated by the TLR-9 agonist CpG generates plasmacytoid dendritic cell (pDC) with enhanced TGF-β and IL-27 production and PD-L1 expression.	Letscher et al., (100)
	EAE in C57BL/6 females	BCG inactivated by extended freeze-drying	Subcutaneous	Attenuates the inflammation systemically and at the CNS level, alleviating EAE. EFD BCG treated mice reduce pro-inflammatory cytokines production (IL-6, IL-1β, TNF-α and IP-10).	Lippens et al., (101)

On the other hand, β-glucans derived from *C. albicans* have been employed as an adjuvant for collagen, resulting in mice arthritis and suggesting that fungal metabolites can contribute exacerbating to autoimmune diseases such as rheumatoid arthritis (RA) (92). Accordingly, SKG mice (prone to autoimmune arthritis) failed to develop the disease under a specific pathogen-free (SPF) environment (91, 102). However, a single administration of fungal β-glucan triggered severe chronic arthritis in SKG mice and transient arthritis in normal mice (91, 102). Moreover, the administration of *C. albicans* β-glucans acts as an adjuvant in the collagen-induced arthritis (CIA) model and induces more severe arthritis (92). In contrast, administering a β-glucan derived from *Aureobasidium pullulans* on the CIA DBA mice model for four weeks markedly reduced arthritis signs in a dose-dependent manner (93). These results suggest that the effect of β-glucan in the case of RA could vary depending on the source of β-glucan and the dose applied. For example, it was shown that β-glucan derived from *Aureobasidium pullulans* can effectively preserve bone mass by an inhibitory effect on osteoclast differentiation and by attenuating the production of pro-inflammatory cytokines (TNF and IL-1β) (103, 104).

Consequently, the encounter with components capable of inducing trained immunity is not always detrimental for autoimmunity (**Table 1**). Training induction in the context of autoimmunity turns out to be complex, and in some of these conditions, it has even been reported to be beneficial by reducing the severity of the symptoms or delaying disease onset. For example, while autoimmunity induces spontaneous IL-17 production and tissue damage, BCG vaccination only induces a primed status of the cells with enhanced secondary pathogen stimulation (105). Indeed, no higher production of these cytokines was seen without second stimulation (105). Consistently with this notion, some studies even reported a beneficial effect of BCG vaccination on autoimmunity (94, 95). As an example, the inoculation with complete Freund’s adjuvant (CFA, which is composed in part by *M. tuberculosis*) into young non-obese diabetic (NOD) mice not only prevented the development of type 1 diabetes (T1D) but can also reverse the early stages of disease (94, 96, 106). This impact was associated with the production of TNF, which selectively killed only disease-causing cells (autoreactive T cells) and allowed pancreas regeneration (96). It was shown that CFA or BCG did not inhibit the development of autoimmunity in mice but redirected the disease from a destructive to a non-destructive process (94). Accordingly, a study in humans using a single dose BCG vaccination reported remission by 4-6 weeks with stabilization of blood sugars in 65% of pre-diabetic patients (94). Another trial concluded that BCG treatment or Epstein-Barr virus (EBV) infection could transiently modify T1D severity in humans by stimulating the innate immune response and suggested that BCG or other stimulators of host innate immunity may contribute to the treatment of long-term diabetes (95). Also, the study proposed that more frequent or higher dosing of BCG will likely be helpful for therapeutic and sustained amelioration of the autoimmunity, based on permanent elimination of autoreactive T cells (95, 107). In addition to the above-mentioned effects, the metabolic changes generated by vaccination with BCG could favor a closer control of blood sugar levels, promoting hypoglycemia (108).

In the case of SSc, it has been shown that macrophages treated with BCG adopt a pro-inflammatory profile, and BCG vaccination in an SSc model exacerbated inflammation (66). Conversely, macrophages from the SSc model exposed to low doses of LPS adopted a profile with lower costimulatory molecules and higher expression of iCOS-ligand and DC-SIGN (mixed M1-M2 phenotype) (66).

Trained immunity has been defined as the altered innate immune response (increased or decreased) to a second encounter, and the mechanisms involved are based on epigenetic and metabolic changes (109). Some components from pathogens, such as helminths, can induce lasting epigenetic changes in stem cells, favoring an enhanced anti-inflammatory response rather than a pro-inflammatory one in the face of a second encounter (98, 99). These reports demonstrate a trained immunity characterized by the enhanced anti-inflammatory response, which poses an exciting therapy that could induce long-term tolerance in the context of autoimmunity. Thus, it has been described that other elements such as helminth-derived compounds (*Fasciola hepatica* total extract or FHTE) are capable of inducing an attenuated form of trained immunity (anti-inflammatory profile) that protects against the induction of the experimental autoimmune encephalomyelitis (EAE), which is the animal model for multiple sclerosis (MS) disease (98). An increased production of IL-10 and IL-1RA by macrophages was observed after FHTE exposure (98). Furthermore, it was recently reported that *F. hepatica* excretory-secretory products (FHES) induce an anti-inflammatory profile in HSCs by increasing the differentiation and proliferation of Lys6Clow monocytes (99). These mice also showed an increased proportion of M2 macrophages and all of these events attenuated and delayed the induction of murine EAE for at least eight months. Even more interesting is the fact that the transfer of HSCs from FHES-treated mice to naive mice transferred this resistance to developing EAE, showing that the effect was both peripheral and central (99). Besides, *in vivo* and *in vitro* stimulation of the TLR-9 in bone marrow induced the migration of precursors of plasmacytoid dendritic cells (pDCs) to the spinal cord and induced the production of TGF-β and IL-27, protecting against the development of EAE (100). On the other hand, at the peripheral level, BCG administration also reduced the severity of EAE in mice by promoting pDCs to induce IL-10-producing Treg cells (101). Although it is unknown how training occurs during autoimmunity, knowing the mechanisms that promote inflammation could lead to new strategies based on metabolic and epigenetic modifications (82).

## Beneficial and Detrimental Effects of Trained Immunity Induction for Autoinflammation

In contrast with the autoimmune diseases triggered by an aberrant adaptive immune response, in autoinflammatory (AIF) diseases, the innate immune response directly induces tissue inflammation in the absence of autoreactive T cells and high autoantibody titers (110, 111). Autoimmune and AIF diseases share some features despite the different key players, such as the prefix “auto” indicating a pathological response against self and the chronic inflammation that develops in genetically predisposed individuals (111). Indeed, AIF diseases are monogenic and multifactorial (polygenic) inflammatory conditions whose heterogeneous symptoms are recurrently associated with fever, as in the periodic fever disorders and episodes of acute inexplicable inflammation (85). In the spectrum of AIF, a subset includes hereditary conditions associated with monogenetic mutations affecting the innate immune system, such as the autosomal dominant TNF receptor-associated periodic syndrome (TRAPS), in which mutations in the genes encoding for the tumor necrosis factor receptor (TNFR1) have been identified (112). In the Hyper IgD syndrome (HIDS), the affecting gene encoding MVK is responsible for increased mevalonic acid, IgD, and IL-1β in the serum (113). Remarkably, mevalonate accumulation is one of the contributors to the induction of trained immunity (26). Another subset of AIF is associated with polygenic mutations and involves several environmental factors, such as Crohn’s disease, Behcet’s disease, and ulcerative colitis (85). In addition, some rheumatic diseases are intermediate autoimmune and AIF settings since they are major histocompatibility complex (MHC)-I associated but mainly autoantibody negative disorders, such as spondyloarthritis (SpA) and other related diseases such uveitis (114).

A critical pathogenetic mechanism in AIF diseases is the dysregulation of the inflammasomes, multiprotein cytoplasmic complexes relevant to innate immunity and inflammatory responses. The main components of inflammasomes are members of the NLR family that detect PAMPs or DAMPs and initiate inflammasome assembly. Thus is induced the proteolytic activation of caspase 1 or 11 and the cleavage and subsequent release of bioactive IL-1β, a key molecule of inflammation and innate immunity (115). Notably, mutations of genes encoding for the components of the proteins involved in the inflammasome (NLRP3) are implicated in the AIF called Cryopyrin-associated periodic syndromes (CAPS) (116).

On the other hand, some AIF diseases are caused by abnormalities of the ubiquitin-proteasome system (UPS), which regulates multiple cellular processes (117). Mutations that cause loss of UPS function in humans lead to a typical type I IFN gene signature and proteasome-associated autoinflammatory syndromes (PRAASs) (118). Although the causes of the induction of sterile inflammation in subjects with PRAAS are still unknown, it is believed that it could be associated with the propagation of endoplasmic reticulum (ER) stress (119). Proteasome defects are known to lead to the retention of misfolded proteins in the ER, leading to inflammation in a pathogen-free setting (120). Another recently described AIF disease related to UPS malfunction is VEXAS (vacuoles, E1 enzyme, X-linked, autoinflammatory, somatic) syndrome (121). Myeloid lineage-restricted somatic mutations of UBA1 (a gene encoding the ubiquitin-activating enzyme 1) characterize VEXAS, leading to inflammation (121). Although the impact of trained immunity in these conditions is unknown to date, it could be assumed that stimuli that establish the capacity of a strengthened response by the innate system could be harmful in these systemic inflammatory diseases.

Aicardi–Goutières syndrome (AGS) is an inherited disease characterized by mutations that produce the accumulation of nucleic acids and ultimately lead to an abnormal IFN response (chronic overproduction of type I IFN) (122). IFN stimulation could induce trained immunity, but chronic exposure to IFN I or IL-1β could cause the HSC pool to become exhausted, in part because of DNA damage caused by replication stress (123). Even though it is still unknown how HSC exhaustion impacts the trained immunity process, it is believed that DNA damage could be influencing renewal capacity and memory in HSCs (124). Remarkably, similar events are described in AGS patients, and it has been reported that they develop AGS during early childhood and many of them after vaccinations or infections (122). Although an association between AGS and trained immunity has not yet been established, it would be a novel approach to study new therapies (124).

Since AIF diseases are characterized by hyperreactivity of the innate immune system, several recent studies have investigated trained immunity in these diseases. Therefore, trained immunity-related signatures such as increased cytokine production, changes in cellular metabolism (mainly increased glycolysis and lactate production in an mTOR/HIF-1α-dependent manner), and epigenetic reprogramming have been analyzed in AIF conditions. Indeed, genetic studies by microarray demonstrated overexpression of IL-1β and IL-1 receptor 1 (IL-1R1) under basal conditions and following LPS stimulation of monocytes of TRAPS compared with controls (125). Another transcriptomic study demonstrated that the treatment with a human anti-IL-1β monoclonal antibody (Canakinumab) reversed the overexpression of inflammatory response genes including IL-1β, suggesting the central role of IL-1β in the TRAPS pathogenesis (126). Also, *in vitro* experiments demonstrated that mTOR contributes to inflammation in TRAPS patients (127), indicating metabolic changes in this AIF disease. In addition, LPS-stimulated peripheral blood monocytes from Behçet’s disease patients produced more TNF than healthy volunteers (128). Enhanced spontaneous and MDP-induced cytokine secretion by monocytes suggested an *in vivo* pre-activation of monocytes in SpA patients under conventional therapy, which was reverted under TNF inhibitor treatment (129). In patients with HIDS, circulating monocytes with a trained immunity phenotype have been detected since accumulated mevalonate amplifies the AKT-mTOR pathway, which in turn induces HIF-1α activation and a shift from oxidative phosphorylation to glycolysis (26).

The Mo/Ma activation depends on epigenetically controlled functional reprogramming to coordinate a proper response. Thus, demethylation of several inflammasome-related molecules has been described in stimulated monocytes and macrophages. Also, the epigenetic changes characterize trained immunity phenotype, and they have been reported in Mo/Ma of patients with several monogenic or complex AIF diseases (130, 131). During macrophage differentiation and monocyte activation, DNA methylation levels of inflammasome-related genes were analyzed in patients with CAPS, an archetypical monogenic AIF syndrome. Monocytes from untreated patients with CAPS undergo more efficient DNA demethylation than those of healthy subjects. Interestingly, patients with CAPS treated with anti-IL-1 drugs display methylation levels similar to those of healthy control subjects (131). Also, when genome‐wide DNA methylation patterns were analyzed in monocytes from 16 male patients with Behçet’s disease and matched healthy controls, 383 CpG sites were differentially methylated between patients and control (only 125 sites in CD4^+^T cells) (132). Furthermore, mevalonate accumulation induces epigenetic changes in HIDS (26).

Trained immunity could be a likely contributor to AIF diseases. Indeed, heat-inactivated *M. tuberculosis* immunization increased spondylitis and arthritis incidence and accelerated the synchronized onset of spondylitis and arthritis in males and females HLA-B27/Huβ2m transgenic rats (133). On the other hand, etanercept (a TNF inhibitor) treatment delayed the appearance of spondylitis and arthritis and suppressed arthritis severity, evidencing a role of TNF and innate immune activation in the induction phase in this SpA animal model (133).

As aforementioned, BCG vaccination aids in inducting trained immunity. Thus, it has been shown that BCG vaccination enhances the antimicrobial response of innate immune cells (assessed by cytokine production capacity), but at the same time downregulates the systemic inflammation as measured by decreased concentrations of pro-inflammatory proteins in the circulation of a large cohort of healthy volunteers (134). This modulatory effect on systemic inflammation may explain some of the beneficial effects of BCG vaccination in inflammatory diseases (134). Inline, BCG vaccination in mice reduces inflammation in murine models of colitis by stimulating IL-10 and TGF-β production and expansion of Tregs (135). Furthermore, BCG decreased mice’s circulating pro-inflammatory cytokines, cholesterol levels, and atherosclerotic lesions [114]. Also, BCG vaccination had a beneficial effect on Alzheimer’s disease, downregulating inflammatory processes (136). In addition, a recent study showed that LPS low-tolerized human macrophages elicit a suppressor effect and mitigate the fibro-inflammatory phenotype of endometriotic cells in an IL-10-dependent manner (97). Although much more needs to be learned, these studies show that the manipulation of trained immunity has therapeutic potential for treating a wide range of hyper-inflammatory conditions.

## Therapies Targeting Trained Immunity in an Autoimmune/Autoinflammatory Context

As we have discussed throughout this review, understanding trained immunity and its detrimental effect on some autoimmune diseases such as SLE or RA (**Table 1**) or AIF have made a call to consider new factors that could increase the severity of these diseases. In this way, PBMC from RA patients compared to healthy individuals shows a different *in vitro* response against BCG extract exposure. Healthy controls produce higher TGF-β and IL-10 levels and lower IFN-γ by BCG stimulation than RA patients, suggesting a tighter regulation in healthy individuals (101). However, there has not been evidence of established trained immunity in monocytes from RA patients, at least concerning the epigenetic alterations in pro-inflammatory genes TNF and IL-6 (137). On the other hand, the relapses in RA and SpA patients are frequent despite bone marrow transplantation. Hence, it has been suggested that transient infections of the bone marrow close to the synovium and entheses (in RA and SpA, respectively) could have induced lasting epigenetic changes in some bone marrow-derived mesenchymal stem cells (BM-MSCs) (138). Furthermore, a trained immunity signature was detected in HSCs in mice with lupus, and in patients, both showed more significant cell proliferation and differentiation, as well as transcriptional activation of cytokines that lead to myelopoiesis (87).

Hence, some therapies propose different strategies to restore or erase the mark of trained immunity to reduce chronic inflammation and tissue destruction. In this sense, we could find drugs that prevent the activation of NOD2 or dectin-1 (GSK669, GSK717, or laminarin) (139) or those that affect the metabolic pathways associated with trained immunity, such as mTOR inhibitors, such as rapamycin (140). Furthermore, trained immunity induced by β-glucan can be inhibited by blocking the rate-limiting enzyme HMG-CoA reductase (reduction of cholesterol synthesis) with fluvastatin *in vitro* (26). Otherwise, another strategy is to modulate epigenetic changes using inhibitors of enzymes that methylate histones or DNA, including DNA methyltransferases, lysine methyltransferases, and histone deacetylases (141). In this sense, the use of nanocarriers that lead the mentioned compounds to a particular cell type (or its progenitors) emerges as a promising alternative to avoid the damaging effects of blocking the indicated pathways (81).

On the other hand, several studies propose (as we have detailed above) that trained immunity can promote a beneficial response in autoimmune conditions by inducing a macrophage response that can result in the apoptosis of active autoreactive T cells or by the promotion of an anti-inflammatory profile (96). Moreover, other reports indicate that by using helminths components (98, 99) or low LPS-dose (66), we could redirect trained immunity to regulate immune responses and reduce chronic inflammation (**Figure 1**). On the other hand, the observation that vaccination (more frequently documented with BCG) could be helpful to treat autoimmune and AIF diseases is encouraging since it has been widely used for many decades worldwide. Interestingly, although the duration of trained immunity has been reported for a few months, and even 1-5 years (39), transgenerational effects have recently been suggested (142, 143). Consequently, these strategies are interesting in the context of chronic diseases, as they promise a long-term beneficial effect.

## Conclusions

This article aimed to review the cellular changes produced during trained immunity and weigh the effect of immune boost in the development/treatment of autoimmune and AIF conditions. As we discuss, components that induce trained immunity aid in prevention and inducing antimicrobial therapy and mitigate some immune-mediated diseases. Thus, the use of stimuli that induce a trained immune response (enhanced or attenuated) may be beneficial in reducing the severity of various autoimmune diseases, as observed in SSc, MS, and T1D models. Along these lines, the consequences of this trained response will depend not only on the nature and concentration of the stimulus but also on the pathologic context (the type of autoimmunity/autoinflammation or infection).

In addition to the vaccination, β-glucans, helminth components, or pharmacological strategies for rebalancing the immune response, it is interesting to mention that other factors such as diet also affect the establishment of trained immunity (144). In this way, a set of environmental factors, such as diet and pollution, may be influencing the long-term immune response profile in the long term. Furthermore, already established “epigenetic scars” can be detected in autoimmunity, which opens the possibility of developing therapies that reverse this scenario (erasure or rewriting in the direction of an attenuated response). These events have been studied for decades in MS and T1D models; however, the warning arises to consider certain factors that can worsen conditions such as SLE and RA in response to some stimuli (**Table 1**).

This article intends to contrapose the controversial evidence concerning how trained immunity may impact autoimmune/autoinflammation conditions. Furthermore, understanding the mechanism of trained immunity raises new immunotherapy strategies aimed at long-term rebalancing immune responses. In this way, one could use the understanding to delete trained immunity marks if it favors the establishment of harmful chronic inflammation or increase it in the situation in which helps lowering the severity of autoimmune diseases.

## Author Contributions

SCF, MR, AF-F, and MDG wrote the manuscript. AK and MDG proofread the manuscript and corrected language use. SCF constructed the figure and table. SCF, MDG, and AK supervised the work and performed critical revision of the manuscript. All authors revised and approved the manuscript.

## Funding

Millennium Institute on Immunology and Immunotherapy grant number P09/016-F, ICN09_016 and ICN2021_045. CORFO grant #13CTI-21526/P4 and P5; ANID/FONDECYT; #1190830 (AK). Biomedical Research Consortium CTU06 (AK). COPEC-UC2019.R.1169. COPECUC2020.E.1. National Agency for Promotion of Science and Technology [PICT-2017-2828], and the National University of San Luis [PROICO-02-1218]. MDG is member of the Scientific Career of the National Council of Scientific and Technical Investigations (CONICET); SCF has a fellowship from the CONICET.

## Conflict of Interest

The authors declare that the research was conducted in the absence of any commercial or financial relationships that could be construed as a potential conflict of interest.

## Publisher’s Note

All claims expressed in this article are solely those of the authors and do not necessarily represent those of their affiliated organizations, or those of the publisher, the editors and the reviewers. Any product that may be evaluated in this article, or claim that may be made by its manufacturer, is not guaranteed or endorsed by the publisher.

## References

[1] Delves PJ Martin SJ Burton DR Roitt IM. Roitt's Essential Immunology. New York: John Wiley & Sons (2006).

[2] Barton ES White DW Cathelyn JS Brett-McClellan KA Engle M Diamond MS. Herpesvirus Latency Confers Symbiotic Protection From Bacterial Infection. Nature (2007) 447(7142):326–9. doi: 10.1038/nature05762 17507983

[3] Sun JC Beilke JN Lanier LL. Immune Memory Redefined: Characterizing the Longevity of Natural Killer Cells. Immunol Rev (2010) 236(1):83–94. doi: 10.1111/j.1600-065X.2010.00900.x 20636810 PMC2907527

[4] Netea MG Quintin J van der Meer JW. Trained Immunity: A Memory for Innate Host Defense. Cell Host Microbe (2011) 9(5):355–61. doi: 10.1016/j.chom.2011.04.006 21575907

[5] Ryals JA Neuenschwander UH Willits MG Molina A Steiner H-Y Hunt MD. Systemic Acquired Resistance. Plant Cell (1996) 8(10):1809. doi: 10.1105/tpc.8.10.1809 12239363 PMC161316

[6] Gourbal B Pinaud S Beckers GJ van der Meer JW Conrath U Netea MG. Innate Immune Memory: An Evolutionary Perspective. Immunol Rev (2018) 283(1):21–40. doi: 10.1111/imr.12647 29664574

[7] Owen AM Fults JB Patil NK Hernandez A Bohannon JK. TLR Agonists as Mediators of Trained Immunity: Mechanistic Insight and Immunotherapeutic Potential to Combat Infection. Front Immunol (2020) 11:3866. doi: 10.3389/fimmu.2020.622614 PMC7930332 33679711

[8] Dominguez-Andres J Netea MG. Long-Term Reprogramming of the Innate Immune System. J Leukocyte Biol (2019) 105(2):329–38. doi: 10.1002/JLB.MR0318-104R 29999546

[9] Chen GY Nuñez G. Sterile Inflammation: Sensing and Reacting to Damage. Nat Rev Immunol (2010) 10(12):826–37. doi: 10.1038/nri2873 PMC3114424 21088683

[10] Mirzakhani M Shahbazi M Shamdani S Naserian S Mohammadnia-Afrouzi M. Innate Immunity: Trained Immunity and Innate Allorecognition Against the Allograft. Int Rev Immunol (2021) 67:1–8. doi: 10.1080/08830185.2021.1921175 33939576

[11] Cheng S-C Scicluna BP Arts RJ Gresnigt MS Lachmandas E Giamarellos-Bourboulis EJ. Broad Defects in the Energy Metabolism of Leukocytes Underlie Immunoparalysis in Sepsis. Nat Immunol (2016) 17(4):406–13. doi: 10.1038/ni.3398 26950237

[12] Pearce EL Pearce EJ. Metabolic Pathways in Immune Cell Activation and Quiescence. Immunity (2013) 38(4):633–43. doi: 10.1016/j.immuni.2013.04.005 PMC3654249 23601682

[13] Newsholme P Gordon S Newsholme EA. Rates of Utilization and Fates of Glucose, Glutamine, Pyruvate, Fatty Acids and Ketone Bodies by Mouse Macrophages. Biochem J (1987) 242(3):631–6. doi: 10.1042/bj2420631 PMC1147758 3593269

[14] Lachmandas E Boutens L Ratter JM Hijmans A Hooiveld GJ Joosten LA. Microbial Stimulation of Different Toll-Like Receptor Signalling Pathways Induces Diverse Metabolic Programmes in Human Monocytes. Nat Microbiol (2016) 2(3):1–10. doi: 10.1038/nmicrobiol.2016.246 27991883

[15] Cheng S-C Quintin J Cramer RA Shepardson KM Saeed S Kumar V. mTOR-And HIF-1α–Mediated Aerobic Glycolysis as Metabolic Basis for Trained Immunity. Science (2014) 345(6204):1250684. doi: 10.1126/science.1250684 25258083 PMC4226238

[16] Arts RJW Carvalho A La Rocca C Palma C Rodrigues F Silvestre R. Immunometabolic Pathways in BCG-Induced Trained Immunity. Cell Rep (2016) 17(10):2562–71. doi: 10.1016/j.celrep.2016.11.011 PMC5177620 27926861

[17] Jung J Zeng H Horng T. Metabolism as a Guiding Force for Immunity. Nat Cell Biol (2019) 21(1):85–93. doi: 10.1038/s41556-018-0217-x 30602764

[18] O’Neill LA Pearce EJ. Immunometabolism Governs Dendritic Cell and Macrophage Function. J Exp Med (2016) 213(1):15–23. doi: 10.1084/jem.20151570 26694970 PMC4710204

[19] Tannahill G Curtis A Adamik J Palsson-McDermott E McGettrick A Goel G. Succinate is an Inflammatory Signal That Induces IL-1β Through HIF-1α. Nature (2013) 496(7444):238–42. doi: 10.1038/nature11986 PMC4031686 23535595

[20] Sun L Yang X Yuan Z Wang H. Metabolic Reprogramming in Immune Response and Tissue Inflammation. Arteriosclerosis thrombosis Vasc Biol (2020) 40(9):1990–2001. doi: 10.1161/ATVBAHA.120.314037 PMC7484156 32698683

[21] Funes SC Rios M Escobar-Vera J Kalergis AM. Implications of Macrophage Polarization in Autoimmunity. Immunology (2018) 154(2):186–95. doi: 10.1111/imm.12910 PMC5980179 29455468

[22] Galván-Peña S O’Neill LA. Metabolic Reprograming in Macrophage Polarization. Front Immunol (2014) 5:420. doi: 10.3389/fimmu.2014.00420 25228902 PMC4151090

[23] Everts B Amiel E Huang SC-C Smith AM Chang C-H Lam WY. TLR-Driven Early Glycolytic Reprogramming *via* the Kinases TBK1-IKKε Supports the Anabolic Demands of Dendritic Cell Activation. Nat Immunol (2014) 15(4):323–32. doi: 10.1038/ni.2833 PMC4358322 24562310

[24] Rodríguez-Espinosa O Rojas-Espinosa O Moreno-Altamirano MMB López-Villegas EO Sánchez-García FJ. Metabolic Requirements for Neutrophil Extracellular Traps Formation. Immunology (2015) 145(2):213–24. doi: 10.1111/imm.12437 PMC4427386 25545227

[25] Keppel MP Saucier N Mah AY Vogel TP Cooper MA. Activation-Specific Metabolic Requirements for NK Cell IFN-γ Production. J Immunol (2015) 194(4):1954–62. doi: 10.4049/jimmunol.1402099 PMC4323953 25595780

[26] Bekkering S Arts RJ Novakovic B Kourtzelis I van der Heijden CD Li Y. Metabolic Induction of Trained Immunity Through the Mevalonate Pathway. Cell (2018) 172(1-2):135–46.e9. doi: 10.1016/j.cell.2017.11.025 29328908

[27] Donohoe DR Bultman SJ. Metaboloepigenetics: Interrelationships Between Energy Metabolism and Epigenetic Control of Gene Expression. J Cell Physiol (2012) 227(9):3169–77. doi: 10.1002/jcp.24054 PMC3338882 22261928

[28] Arts RJ Blok BA Aaby P Joosten LA de Jong D van der Meer JW. Long-Term *In Vitro* and *In Vivo* Effects of γ-Irradiated BCG on Innate and Adaptive Immunity. J Leukocyte Biol (2015) 98(6):995–1001. doi: 10.1189/jlb.4MA0215-059R 26082519

[29] Schvartzman JM Thompson CB Finley LW. Metabolic Regulation of Chromatin Modifications and Gene Expression. J Cell Biol (2018) 217(7):2247–59. doi: 10.1083/jcb.201803061 PMC6028552 29760106

[30] Netea MG Joosten LA Latz E Mills KH Natoli G Stunnenberg HG. Trained immunity: a program of innate immune memory in health and disease. Science (2016) 352(6284):aaf1098. doi: 10.1126/science.aaf1098 27102489 PMC5087274

[31] Arts RJW Novakovic B ter Horst R Carvalho A Bekkering S Lachmandas E. Glutaminolysis and Fumarate Accumulation Integrate Immunometabolic and Epigenetic Programs in Trained Immunity. Cell Metab (2016) 24(6):807–19. doi: 10.1016/j.cmet.2016.10.008 PMC5742541 27866838

[32] Domínguez-Andrés J Novakovic B Li Y Scicluna BP Gresnigt MS Arts RJ. The Itaconate Pathway is a Central Regulatory Node Linking Innate Immune Tolerance and Trained Immunity. Cell Metab (2019) 29(1):211–20.e5. doi: 10.1016/j.cmet.2018.09.003 30293776

[33] Funes SC Fernández-Fierro A Rebolledo-Zelada D Mackern-Oberti JP Kalergis AM. Contribution of Dysregulated DNA Methylation to Autoimmunity. Int J Mol Sci (2021) 22(21):11892. doi: 10.3390/ijms222111892 34769338 PMC8584328

[34] Chen S Yang J Wei Y Wei X. Epigenetic Regulation of Macrophages: From Homeostasis Maintenance to Host Defense. Cell Mol Immunol (2020) 17(1):36–49. doi: 10.1038/s41423-019-0315-0 31664225 PMC6952359

[35] Quintin J Saeed S Martens JH Giamarellos-Bourboulis EJ Ifrim DC Logie C. Candida Albicans Infection Affords Protection Against Reinfection *via* Functional Reprogramming of Monocytes. Cell Host Microbe (2012) 12(2):223–32. doi: 10.1016/j.chom.2012.06.006 PMC3864037 22901542

[36] Ifrim DC Quintin J Joosten LA Jacobs C Jansen T Jacobs L. Trained Immunity or Tolerance: Opposing Functional Programs Induced in Human Monocytes After Engagement of Various Pattern Recognition Receptors. Clin Vaccine Immunol (2014) 21(4):534–45. doi: 10.1128/CVI.00688-13 PMC3993125 24521784

[37] Foster SL Hargreaves DC Medzhitov R. Gene-Specific Control of Inflammation by TLR-Induced Chromatin Modifications. Nature (2007) 447(7147):972–8. doi: 10.1038/nature05836 17538624

[38] Novakovic B Habibi E Wang S-Y Arts RJW Davar R Megchelenbrink W. β-Glucan Reverses the Epigenetic State of LPS-Induced Immunological Tolerance. Cell (2016) 167(5):1354–68.e14. doi: 10.1016/j.cell.2016.09.034 27863248 PMC5927328

[39] Nankabirwa V Tumwine JK Mugaba PM Tylleskär T Sommerfelt H. Child Survival and BCG Vaccination: A Community Based Prospective Cohort Study in Uganda. BMC Public Health (2015) 15(1):1–10. doi: 10.1186/s12889-015-1497-8 25886062 PMC4342809

[40] Kaufmann E Sanz J Dunn JL Khan N Mendonça LE Pacis A. BCG Educates Hematopoietic Stem Cells to Generate Protective Innate Immunity Against Tuberculosis. Cell (2018) 172(1-2):176–90.e19. doi: 10.1016/j.cell.2017.12.031 29328912

[41] Mitroulis I Ruppova K Wang B Chen L-S Grzybek M Grinenko T. Modulation of Myelopoiesis Progenitors is an Integral Component of Trained Immunity. Cell (2018) 172(1-2):147–61. e12. doi: 10.1016/j.cell.2017.11.034 29328910 PMC5766828

[42] Cirovic B de Bree LCJ Groh L Blok BA Chan J van der Velden WJ. BCG Vaccination in Humans Elicits Trained Immunity *via* the Hematopoietic Progenitor Compartment. Cell Host Microbe (2020) 28(2):322–34. e5. doi: 10.1016/j.chom.2020.05.014 32544459 PMC7295478

[43] Moorlag SJ Khan N Novakovic B Kaufmann E Jansen T van Crevel R. β-Glucan Induces Protective Trained Immunity Against Mycobacterium Tuberculosis Infection: A Key Role for IL-1. Cell Rep (2020) 31(7):107634. doi: 10.1016/j.celrep.2020.107634 32433977 PMC7242907

[44] Biton M Haber AL Rogel N Burgin G Beyaz S Schnell A. T Helper Cell Cytokines Modulate Intestinal Stem Cell Renewal and Differentiation. Cell (2018) 175(5):1307–20.e22. doi: 10.1016/j.cell.2018.10.008 30392957 PMC6239889

[45] de Laval B Maurizio J Kandalla PK Brisou G Simonnet L Huber C. C/Ebpβ-Dependent Epigenetic Memory Induces Trained Immunity in Hematopoietic Stem Cells. Cell Stem Cell (2020) 26(5):657–74.e8. doi: 10.1016/j.stem.2020.01.017 32169166

[46] Soto JA Gálvez NM Andrade CA Ramírez MA Riedel CA Kalergis AM. BCG Vaccination Induces Cross-Protective Immunity Against Pathogenic Microorganisms. Trends Immunol (2022) 43:322–35. doi: 10.1016/j.it.2021.12.006 35074254

[47] Covián C Ríos M Berríos-Rojas RV Bueno SM Kalergis AM. Induction of Trained Immunity by Recombinant Vaccines. Front Immunol (2021) 3406. doi: 10.3389/fimmu.2020.611946 PMC7873984 33584692

[48] Kleinnijenhuis J Quintin J Preijers F Joosten LA Jacobs C Xavier RJ. BCG-Induced Trained Immunity in NK Cells: Role for non-Specific Protection to Infection. Clin Immunol (2014) 155(2):213–9. doi: 10.1016/j.clim.2014.10.005 PMC5084088 25451159

[49] Acevedo OA Berrios RV Rodríguez-Guilarte L Lillo-Dapremont B Kalergis AM. Molecular and Cellular Mechanisms Modulating Trained Immunity by Various Cell Types in Response to Pathogen Encounter. Front Immunol (2021) 4082. doi: 10.3389/fimmu.2021.745332 PMC8521023 34671359

[50] Gyssens I Netea M. Heterologous Effects of Vaccination and Trained Immunity. Clin Microbiolo Infect (2019) 25(12):1457–8. doi: 10.1016/j.cmi.2019.05.024 31158520

[51] Palgen J-L Feraoun Y Dzangué-Tchoupou G Joly C Martinon F Le Grand R. Optimize Prime/Boost Vaccine Strategies: Trained Immunity as a New Player in the Game. Front Immunol (2021) 12:554. doi: 10.3389/fimmu.2021.612747 PMC7982481 33763063

[52] Garly M-L Martins CL Balé C Baldé MA Hedegaard KL Gustafson P. BCG Scar and Positive Tuberculin Reaction Associated With Reduced Child Mortality in West Africa: A non-Specific Beneficial Effect of BCG? Vaccine (2003) 21(21-22):2782–90. doi: 10.1016/S0264-410X(03)00181-6 12798618

[53] Covián C Retamal-Díaz A Bueno SM Kalergis AM. Could BCG Vaccination Induce Protective Trained Immunity for SARS-CoV-2? Front Immunol (2020) 11:970. doi: 10.3389/fimmu.2020.00970 32574258 PMC7227382

[54] Tribouley J Tribouley-Duret J Appriou M. Effect of Bacillus Callmette Guerin (BCG) on the Receptivity of Nude Mice to Schistosoma Mansoni. Comptes rendus Des seances la Societe biologie ses filiales (1978) 172(5):902–4. 157204

[55] Moorlag S Arts R Van Crevel R Netea M. Non-Specific Effects of BCG Vaccine on Viral Infections. Clin Microbiol infection (2019) 25(12):1473–8. doi: 10.1016/j.cmi.2019.04.020 31055165

[56] Kleinnijenhuis J Quintin J Preijers F Joosten L Ifrim D Saeed S. Bacille Calmette-Guerin Induces NOD2-Dependent Nonspecific Protection From Reinfection *via* Epigenetic Reprogramming of Monocytes. Proc Natl Acad Sci (2012) 109(43):17537–42. doi: 10.1073/pnas.1202870109 PMC3491454 22988082

[57] Aaby P Martins CL Garly M-L Balé C Andersen A Rodrigues A. Non-Specific Effects of Standard Measles Vaccine at 4.5 and 9 Months of Age on Childhood Mortality: Randomised Controlled Trial. Bmj (2010) 341:c6495. doi: 10.1136/bmj.c6495 21118875 PMC2994348

[58] Rieckmann A Villumsen M Jensen ML Ravn H da Silva ZJ Sørup S. The Effect of Smallpox and Bacillus Calmette-Guérin Vaccination on the Risk of Human Immunodeficiency Virus-1 Infection in Guinea-Bissau and Denmark. Open Forum Infect Dis (2017) 4:1–10. doi: 10.1093/ofid/ofx130 PMC5569962 28852677

[59] Kölmel K Grange J Krone B Mastrangelo G Rossi C Henz B. Prior Immunisation of Patients With Malignant Melanoma With Vaccinia or BCG is Associated With Better Survival. An European Organization for Research and Treatment of Cancer Cohort Study on 542 Patients. Eur J Cancer (2005) 41(1):118–25. doi: 10.1016/j.ejca.2004.09.023 15617996

[60] Upfill-Brown A Taniuchi M Platts-Mills JA Kirkpatrick B Burgess SL Oberste MS. Nonspecific Effects of Oral Polio Vaccine on Diarrheal Burden and Etiology Among Bangladeshi Infants. Clin Infect Dis (2017) 65(3):414–9. doi: 10.1093/cid/cix354 PMC5848225 28444240

[61] Lund N Andersen A Hansen ASK Jepsen FS Barbosa A Biering-Sørensen S. The Effect of Oral Polio Vaccine at Birth on Infant Mortality: A Randomized Trial. Clin Infect Diseases (2015) 61(10):1504–11. doi: 10.1093/cid/civ617 PMC4614411 26219694

[62] Tarancón R Domínguez-Andrés J Uranga S Ferreira AV Groh LA Domenech M. New Live Attenuated Tuberculosis Vaccine MTBVAC Induces Trained Immunity and Confers Protection Against Experimental Lethal Pneumonia. PloS Pathogens (2020) 16(4):e1008404. doi: 10.1371/journal.ppat.1008404 32240273 PMC7117655

[63] Girardin SE Boneca IG Viala J Chamaillard M Labigne A Thomas G. Nod2 is a General Sensor of Peptidoglycan Through Muramyl Dipeptide (MDP) Detection. J Biol Chem (2003) 278(11):8869–72. doi: 10.1074/jbc.C200651200 12527755

[64] Guha M Mackman N. LPS Induction of Gene Expression in Human Monocytes. Cell Signal (2001) 13:85–94. doi: 10.1016/S0898-6568(00)00149-2 11257452

[65] Feng Y-w Wu C Liang F-y Lin T Li W-q Jing Y-h. hUCMSCs Mitigate LPS-Induced Trained Immunity in Ischemic Stroke. Front Immunol (2020) 11. doi: 10.3389/fimmu.2020.01746 PMC7516337 33013828

[66] Jeljeli M Riccio LGC Doridot L Chêne C Nicco C Chouzenoux S. Trained Immunity Modulates Inflammation-Induced Fibrosis. Nat Commun (2019) 10(1):1–15. doi: 10.1038/s41467-019-13636-x 31827093 PMC6906311

[67] Seeley JJ Ghosh S. Molecular Mechanisms of Innate Memory and Tolerance to LPS. J Leukocyte Biol (2017) 101(1):107–19. doi: 10.1189/jlb.3MR0316-118RR 27780875

[68] Van Der Valk F Bekkering S Kroon J Yeang C Van Den Bossche J Van Buul J. Oxidized Phospholipids on Lipoprotein (a) Elicit Arterial Wall Inflammation and an Inflammatory Monocyte Response in Humans. Circulation (2016) 134:611–24. doi: 10.1161/CIRCULATIONAHA.116.020838 PMC4995139 27496857

[69] Groh LA Ferreira AV Helder L van der Heijden CD Novakovic B van de Westerlo E. oxLDL-Induced Trained Immunity Is Dependent on Mitochondrial Metabolic Reprogramming. Immunometabolism (2021) 3(3):e210025. doi: 10.20900/immunometab20210025 34267957 PMC7611242

[70] Drummer CI Saaoud F Sun Y Atar D Xu K Lu Y. Hyperlipidemia May Synergize With Hypomethylation in Establishing Trained Immunity and Promoting Inflammation in NASH and NAFLD. J Immunol Res (2021) 2021:3928323. doi: 10.1155/2021/3928323 34859106 PMC8632388

[71] Pirillo A Bonacina F Norata GD Catapano AL. The Interplay of Lipids, Lipoproteins, and Immunity in Atherosclerosis. Curr Atheroscl Rep (2018) 20(3):1–9. doi: 10.1007/s11883-018-0715-0 29445885

[72] van der Heijden C Deinum J Joosten LAB Netea MG Riksen NP. The Mineralocorticoid Receptor as a Modulator of Innate Immunity and Atherosclerosis. Cardiovasc Res (2018) 114(7):944–53. doi: 10.1093/cvr/cvy092 29668907

[73] Sohrabi Y Sonntag GV Braun LC Lagache SM Liebmann M Klotz L. LXR Activation Induces a Proinflammatory Trained Innate Immunity-Phenotype in Human Monocytes. Front Immunol (2020) 11:353. doi: 10.3389/fimmu.2020.00353 32210962 PMC7077358

[74] Vetvicka V Vetvickova J. Anti-Infectious and Anti-Tumor Activities of β-Glucans. Anticancer Res (2020) 40(6):3139–45. doi: 10.21873/anticanres.14295 32487608

[75] Marakalala MJ Williams DL Hoving JC Engstad R Netea MG Brown GD. Dectin-1 Plays a Redundant Role in the Immunomodulatory Activities of β-Glucan-Rich Ligands In Vivo. Microbes Infect (2013) 15(6-7):511–5. doi: 10.1016/j.micinf.2013.03.002 PMC3839404 23518266

[76] Stothers CL Burelbach KR Owen AM Patil NK McBride MA Bohannon JK. β-Glucan Induces Distinct and Protective Innate Immune Memory in Differentiated Macrophages. J Immunol (2021) 207(11):2785–98. doi: 10.4049/jimmunol.2100107 PMC8612974 34740960

[77] Hole CR Wager CML Castro-Lopez N Campuzano A Cai H Wozniak KL. Induction of Memory-Like Dendritic Cell Responses In Vivo. Nat Commun (2019) 10(1):1–13. doi: 10.1038/s41467-019-10486-5 31273203 PMC6609631

[78] Brown GD Herre J Williams DL Willment JA Marshall AS Gordon S. Dectin-1 Mediates the Biological Effects of β-Glucans. J Exp Med (2003) 197(9):1119–24. doi: 10.1084/jem.20021890 PMC2193964 12719478

[79] Saeed S Quintin J Kerstens HH Rao NA Aghajanirefah A Matarese F. Epigenetic Programming of Monocyte-to-Macrophage Differentiation and Trained Innate Immunity. Science (2014) 345(6204):1251086. doi: 10.1126/science.1251086 25258085 PMC4242194

[80] Chavakis T Mitroulis I Hajishengallis G. Hematopoietic Progenitor Cells as Integrative Hubs for Adaptation to and Fine-Tuning of Inflammation. Nat Immunol (2019) 20(7):802–11. doi: 10.1038/s41590-019-0402-5 PMC6709414 31213716

[81] Mulder WJ Ochando J Joosten LA Fayad ZA Netea MG. Therapeutic Targeting of Trained Immunity. Nat Rev Drug Discov (2019) 18(7):553–66. doi: 10.1038/s41573-019-0025-4 PMC7069501 30967658

[82] Arts RJ Joosten LA Netea MG. The Potential Role of Trained Immunity in Autoimmune and Autoinflammatory Disorders. Front Immunol (2018) 9:298. doi: 10.3389/fimmu.2018.00298 29515591 PMC5826224

[83] Toubi E Vadasz Z. Innate Immune-Responses and Their Role in Driving Autoimmunity. Autoimmun Rev (2019) 18(3):306–11. doi: 10.1016/j.autrev.2018.10.005 30639645

[84] Funes SC Ríos M Gómez-Santander F Fernández-Fierro A Altamirano-Lagos MJ Rivera-Perez D. Tolerogenic Dendritic Cell Transfer Ameliorates Systemic Lupus Erythematosus in Mice. Immunology (2019) 158(4):322–39. doi: 10.1111/imm.13119 PMC6856940 31509246

[85] Arakelyan A Nersisyan L Poghosyan D Khondkaryan L Hakobyan A Löffler-Wirth H. Autoimmunity and Autoinflammation: A Systems View on Signaling Pathway Dysregulation Profiles. PloS One (2017) 12(11):e0187572. doi: 10.1371/journal.pone.0187572 29099860 PMC5669448

[86] Herrada AA Llanos C Mackern-Oberti JP Carreño LJ Henriquez C Gómez RS. Haem Oxygenase 1 Expression is Altered in Monocytes From Patients With Systemic Lupus Erythematosus. Immunology (2012) 136(4):414–24. doi: 10.1111/j.1365-2567.2012.03598.x PMC3401980 22587389

[87] Grigoriou M Banos A Filia A Pavlidis P Giannouli S Karali V. Transcriptome Reprogramming and Myeloid Skewing in Haematopoietic Stem and Progenitor Cells in Systemic Lupus Erythematosus. Ann Rheumatic Dis (2019) 79:242–53. doi: 10.1136/annrheumdis-2019-215782 PMC7025734 31780527

[88] Municio C Criado G. Therapies Targeting Trained Immune Cells in Inflammatory and Autoimmune Diseases. Front Immunol (2020) 11. doi: 10.3389/fimmu.2020.631743 PMC7868395 33569065

[89] Saithong S Saisorn W Visitchanakun P Sae-Khow K Chiewchengchol D Leelahavanichkul A. A Synergy Between Endotoxin and (1→ 3)-Beta-D-Glucan Enhanced Neutrophil Extracellular Traps in Candida Administered Dextran Sulfate Solution Induced Colitis in FcGRIIB-/-Lupus Mice, an Impact of Intestinal Fungi in Lupus. J Inflamm Res (2021) 14:2333. doi: 10.2147/JIR.S305225 34103965 PMC8179808

[90] Fagone P Mangano K Mammana S Quattrocchi C Magro G Coco M. Acceleration of SLE-Like Syndrome Development in NZBxNZW F1 Mice by Beta-Glucan. Lupus (2014) 23(4):407–11. doi: 10.1177/0961203314522333 24493283

[91] Yoshitomi H Sakaguchi N Kobayashi K Brown GD Tagami T Sakihama T. A Role for Fungal β-Glucans and Their Receptor Dectin-1 in the Induction of Autoimmune Arthritis in Genetically Susceptible Mice. J Exp Med (2005) 201(6):949–60. doi: 10.1084/jem.20041758 PMC2213107 15781585

[92] Hida S Miura NN Adachi Y Ohno N. Effect of Candida Albicans Cell Wall Glucan as Adjuvant for Induction of Autoimmune Arthritis in Mice. J Autoimmunity (2005) 25(2):93–101. doi: 10.1016/j.jaut.2005.06.002 16242302

[93] Kim J-W Cho H-R Kim K-Y S-k Ku Lee H-S. Effect of Beta-Glucan on the Collagen-Induced Rheumatoid Arthritis. J Veterinary Clinics (2010) 27(4):315–24.

[94] Shehadeh N Calcinaro F Bradley BJ Bruchim I Vardi P Lafferty KJ. Effect of Adjuvant Therapy on Development of Diabetes in Mouse and Man. Lancet (London England) (1994) 343(8899):706–7. doi: 10.1016/S0140-6736(94)91583-0 7907682

[95] Faustman DL Wang L Okubo Y Burger D Ban L Man G. Proof-Of-Concept, Randomized, Controlled Clinical Trial of Bacillus-Calmette-Guerin for Treatment of Long-Term Type 1 Diabetes. Plos One (2012) 7(8):e41756. doi: 10.1371/journal.pone.0041756 22905105 PMC3414482

[96] Ryu S Kodama S Ryu K Schoenfeld DA Faustman DL. Reversal of Established Autoimmune Diabetes by Restoration of Endogenous β Cell Function. J Clin Invest (2001) 108(1):63–72. doi: 10.1172/JCI12335 11435458 PMC209340

[97] Jeljeli M Riccio LG Chouzenoux S Moresi F Toullec L Doridot L. Macrophage Immune Memory Controls Endometriosis in Mice and Humans. Cell Rep (2020) 33(5):108325. doi: 10.1016/j.celrep.2020.108325 33147452

[98] Quinn SM Cunningham K Raverdeau M Walsh RJ Curham L Malara A. Anti-Inflammatory Trained Immunity Mediated by Helminth Products Attenuates the Induction of T Cell-Mediated Autoimmune Disease. Front Immunol (2019) 10:1109. doi: 10.3389/fimmu.2019.01109 31178861 PMC6537856

[99] Cunningham KT Finlay CM Mills KH. Helminth Imprinting of Hematopoietic Stem Cells Sustains Anti-Inflammatory Trained Innate Immunity That Attenuates Autoimmune Disease. J Immunol (2021) 206(7):1618–30. doi: 10.4049/jimmunol.2001225 33579723

[100] Letscher H Agbogan VA Korniotis S Gastineau P Tejerina E Gras C. Toll-Like Receptor-9 Stimulated Plasmacytoid Dendritic Cell Precursors Suppress Autoimmune Neuroinflammation in a Murine Model of Multiple Sclerosis. Sci Rep (2021) 11(1):1–17. doi: 10.1038/s41598-021-84023-0 33637789 PMC7910458

[101] Lippens C Garnier L Guyonvarc'h P-M Santiago-Raber M-L Hugues S. Extended Freeze-Dried BCG Instructed pDCs Induce Suppressive Tregs and Dampen EAE. Front Immunol (2018) 9:2777. doi: 10.3389/fimmu.2018.02777 30555468 PMC6281986

[102] Hida S Miura NN Adachi Y Ohno N. Cell Wall β-Glucan Derived From Candida Albicans Acts as a Trigger for Autoimmune Arthritis in SKG Mice. Biol Pharm Bulletin (2007) 30(8):1589–92. doi: 10.1248/bpb.30.1589 17666828

[103] Jung MY Kim JW Kim KY Choi SH Ku SK. Polycan, a β-Glucan From Aureobasidium Pullulans SM-2001, Mitigates Ovariectomy-Induced Osteoporosis in Rats. Exp Ther Med (2016) 12(3):1251–62. doi: 10.3892/etm.2016.3485 PMC4998138 27588046

[104] Kim Y Kang S Kim J Cho H Moon S Kim K. Effects of Polycan, a β-Glucan, on Experimental Periodontitis and Alveolar Bone Loss in Sprague-Dawley Rats. J periodontal Res (2012) 47(6):800–10. doi: 10.1111/j.1600-0765.2012.01502.x 22780690

[105] Kleinnijenhuis J Quintin J Preijers F Benn CS Joosten LA Jacobs C. Long-Lasting Effects of BCG Vaccination on Both Heterologous Th1/Th17 Responses and Innate Trained Immunity. J Innate Immun (2014) 6(2):152–8. doi: 10.1159/000355628 PMC3944069 24192057

[106] Kodama S Kühtreiber W Fujimura S Dale EA Faustman DL. Islet Regeneration During the Reversal of Autoimmune Diabetes in NOD Mice. Science (2003) 302(5648):1223–7. doi: 10.1126/science.1088949 14615542

[107] Faustman DL. TNF. TNF Inducers, and TNFR2 Agonists: A New Path to Type 1 Diabetes Treatment. Diabetes/Metabolism Res Rev (2018) 34(1):e2941. doi: 10.1002/dmrr.2941 28843039

[108] Faustman D. Benefits of BCG-Induced Metabolic Switch From Oxidative Phosphorylation to Aerobic Glycolysis in Autoimmune and Nervous System Diseases. J Internal Med (2020) 288(6):641–50. doi: 10.1111/joim.13050 32107806

[109] Netea MG Domínguez-Andrés J Barreiro LB Chavakis T Divangahi M Fuchs E. Defining Trained Immunity and its Role in Health and Disease. Nat Rev Immunol (2020) 20(6):375–88. doi: 10.1038/s41577-020-0285-6 PMC7186935 32132681

[110] Szekanecz Z McInnes IB Schett G Szamosi S Benkő S Szűcs G. Autoinflammation and Autoimmunity Across Rheumatic and Musculoskeletal Diseases. Nat Rev Rheumatol (2021) 17:1–11. doi: 10.1038/s41584-021-00652-9 34341562

[111] Doherty TA Brydges SD Hoffman HM. Autoinflammation: Translating Mechanism to Therapy. J leukocyte Biol (2011) 90(1):37–47. doi: 10.1189/jlb.1110616 21330349 PMC3219035

[112] Melek M Gellert M. RAG1/2-Mediated Resolution of Transposition Intermediates: Two Pathways and Possible Consequences. Cell (2000) 101(6):625–33. doi: 10.1016/S0092-8674(00)80874-0 10892649

[113] Houten SM Kuis W Duran M De Koning TJ van Royen-Kerkhof A Romeijn GJ. Mutations in MVK, Encoding Mevalonate Kinase, Cause Hyperimmunoglobulinaemia D and Periodic Fever Syndrome. Nat Genet (1999) 22(2):175–7. doi: 10.1038/9691 10369261

[114] Zen M Gatto M Domeneghetti M Palma L Borella E Iaccarino L. Clinical Guidelines and Definitions of Autoinflammatory Diseases: Contrasts and Comparisons With Autoimmunity—a Comprehensive Review. Clin Rev Allergy Immunol (2013) 45(2):227–35. doi: 10.1007/s12016-013-8355-1 23322404

[115] Havnaer A Han G. Autoinflammatory Disorders: A Review and Update on Pathogenesis and Treatment. Am J Clin Dermatol (2019) 20(4):539–64. doi: 10.1007/s40257-019-00440-y 30997665

[116] Mortimer L Moreau F MacDonald JA Chadee K. NLRP3 Inflammasome Inhibition is Disrupted in a Group of Auto-Inflammatory Disease CAPS Mutations. Nat Immunol (2016) 17(10):1176–86. doi: 10.1038/ni.3538 27548431

[117] Çetin G Klafack S Studencka-Turski M Krüger E Ebstein F. The Ubiquitin–Proteasome System in Immune Cells. Biomolecules (2021) 11(1):60. doi: 10.3390/biom11010060 33466553 PMC7824874

[118] Goetzke CC Ebstein F Kallinich T. Role of Proteasomes in Inflammation. J Clin Med (2021) 10(8):1783. doi: 10.3390/jcm10081783 33923887 PMC8072576

[119] Obeng EA Carlson LM Gutman DM Harrington WJ Jr. Lee KP Boise LH. Proteasome Inhibitors Induce a Terminal Unfolded Protein Response in Multiple Myeloma Cells. Blood (2006) 107(12):4907–16. doi: 10.1182/blood-2005-08-3531 PMC1895817 16507771

[120] Ebstein F Poli Harlowe MC Studencka-Turski M Krüger E. Contribution of the Unfolded Protein Response (UPR) to the Pathogenesis of Proteasome-Associated Autoinflammatory Syndromes (PRAAS). Front Immunol (2019) 10:2756. doi: 10.3389/fimmu.2019.02756 31827472 PMC6890838

[121] Beck DB Ferrada MA Sikora KA Ombrello AK Collins JC Pei W. Somatic Mutations in UBA1 and Severe Adult-Onset Autoinflammatory Disease. N Engl J Med (2020) 383(27):2628–38. doi: 10.1056/NEJMoa2026834 PMC7847551 33108101

[122] Rice GI Kitabayashi N Barth M Briggs TA Burton AC Carpanelli ML. Genetic, Phenotypic, and Interferon Biomarker Status in ADAR1-Related Neurological Disease. Neuropediatrics (2017) 48(03):166–84. doi: 10.1055/s-0037-1601449 PMC5985975 28561207

[123] Visan I. Stressed HSCs. Nat Immunol (2015) 16(4):342. doi: 10.1038/ni.3138

[124] Chen L Ozato K. Innate Immune Memory in Hematopoietic Stem/Progenitor Cells: Myeloid-Biased Differentiation and the Role of Interferon. Front Immunol (2021) 12:1005. doi: 10.3389/fimmu.2021.621333 PMC8039377 33854500

[125] Borghini S Ferrera D Prigione I Fiore M Ferraris C Mirisola V. Gene Expression Profile in TNF Receptor-Associated Periodic Syndrome Reveals Constitutively Enhanced Pathways and New Players in the Underlying Inflammation. Clin Exp Rheumatol (2016) 34(6 Suppl 102):S121–S8. 27310036

[126] Torene R Nirmala N Obici L Cattalini M Tormey V Caorsi R. Canakinumab Reverses Overexpression of Inflammatory Response Genes in Tumour Necrosis Factor Receptor-Associated Periodic Syndrome. Ann rheumatic diseases (2017) 76(1):303–9. doi: 10.1136/annrheumdis-2016-209335 PMC5264306 27474763

[127] Bachetti T Chiesa S Castagnola P Bani D Di Zanni E Omenetti A. Autophagy Contributes to Inflammation in Patients With TNFR-Associated Periodic Syndrome (TRAPS). Ann Rheumatic Dis (2013) 72(6):1044–52. doi: 10.1136/annrheumdis-2012-201952 23117241

[128] Slobodin G Toukan Y Rosner I Rozenbaum M Boulman N Pavlotzky E. LPS-Stimulated Production of TNF-α by Peripheral Blood Monocytes in Patients With Behcet’s Disease. Clin Rheumatol (2007) 26(5):764. doi: 10.1007/s10067-006-0371-6 16897113

[129] Conrad K Wu P Sieper J Syrbe U. *In Vivo* Pre-Activation of Monocytes in Patients With Axial Spondyloarthritis. Arthritis Res Ther (2015) 17(1):1–12. doi: 10.1186/s13075-015-0694-2 26178906 PMC4504100

[130] Kirectepe AK Kasapcopur O Arisoy N Erdem GC Hatemi G Ozdogan H. Analysis of MEFV Exon Methylation and Expression Patterns in Familial Mediterranean Fever. BMC Med Genet (2011) 12(1):1–6. doi: 10.1186/1471-2350-12-105 21819621 PMC3175150

[131] Vento-Tormo R Álvarez-Errico D Garcia-Gomez A Hernández-Rodríguez J Buján S Basagaña M. DNA Demethylation of Inflammasome-Associated Genes is Enhanced in Patients With Cryopyrin-Associated Periodic Syndromes. J Allergy Clin Immunol (2017) 139(1):202–11.e6. doi: 10.1016/j.jaci.2016.05.016 27394913

[132] Hughes T Ture-Ozdemir F Alibaz-Oner F Coit P Direskeneli H Sawalha AH. Epigenome-Wide Scan Identifies a Treatment-Responsive Pattern of Altered DNA Methylation Among Cytoskeletal Remodeling Genes in Monocytes and CD4+ T Cells From Patients With Behcet's Disease. Arthritis Rheumatol (2014) 66(6):1648–58. doi: 10.1002/art.38409 PMC4096298 24574333

[133] van Tok MN Satumtira N Dorris M Pots D Slobodin G van de Sande MG. Innate Immune Activation can Trigger Experimental Spondyloarthritis in HLA-B27/Huβ2m Transgenic Rats. Front Immunol (2017) 8:920. doi: 10.3389/fimmu.2017.00920 28824645 PMC5545590

[134] Koeken VA de Bree LCJ Mourits VP Moorlag SJ Walk J Cirovic B. BCG Vaccination in Humans Inhibits Systemic Inflammation in a Sex-Dependent Manner. J Clin Invest (2020) 130(10):5591–602. doi: 10.1172/JCI133935 PMC7524503 32692728

[135] Lagranderie M Kluge C Kiefer–Biasizzo H Abolhassani M Nahori MA Fitting C. Mycobacterium Bovis Bacillus Calmette-Guerin Killed by Extended Freeze-Drying Reduces Colitis in Mice. Gastroenterology (2011) 141(2):642–52.e4. doi: 10.1053/j.gastro.2011.05.002 21683076

[136] Ovchinnikova O Berge N Kang C Urien C Ketelhuth D Pottier J. Mycobacterium Bovis BCG Killed by Extended Freeze-Drying Induces an Immunoregulatory Profile and Protects Against Atherosclerosis. J Internal Med (2014) 275(1):49–58. doi: 10.1111/joim.12127 23962000

[137] Messemaker T Mikkers H Huizinga T Toes R van der Helm-Van Mil A Kurreeman F. Inflammatory Genes Tnfα and IL6 Display No Signs of Increased H3K4me3 in Circulating Monocytes From Untreated Rheumatoid Arthritis Patients. Genes Immunity (2017) 18(3):191–6. doi: 10.1038/gene.2017.20 28794503

[138] Berthelot J-M Le Goff B Maugars Y. Bone Marrow Mesenchymal Stem Cells in Rheumatoid Arthritis, Spondyloarthritis, and Ankylosing Spondylitis: Problems Rather Than Solutions? Arthritis Res Ther (2019) 21(1):1–9. doi: 10.1186/s13075-019-2014-8 31722720 PMC6854713

[139] Rickard DJ Sehon CA Kasparcova V Kallal LA Zeng X Montoute MN. Identification of Benzimidazole Diamides as Selective Inhibitors of the Nucleotide-Binding Oligomerization Domain 2 (NOD2) Signaling Pathway. PloS One (2013) 8(8):e69619. doi: 10.1371/journal.pone.0069619 23936340 PMC3731320

[140] Dowling RJ Topisirovic I Fonseca BD Sonenberg N. Dissecting the Role of mTOR: Lessons From mTOR Inhibitors. Biochim Biophys Acta (BBA)-Proteins Proteomics (2010) 1804(3):433–9. doi: 10.1016/j.bbapap.2009.12.001 20005306

[141] Tough DF Tak PP Tarakhovsky A Prinjha RK. Epigenetic Drug Discovery: Breaking Through the Immune Barrier. Nat Rev Drug Discov (2016) 15(12):835–53. doi: 10.1038/nrd.2016.185 27765940

[142] Berendsen MLT Øland CB Bles P Jensen AKG Kofoed P-E Whittle H. Maternal Priming: Bacillus Calmette-Guérin (BCG) Vaccine Scarring in Mothers Enhances the Survival of Their Child With a BCG Vaccine Scar. J Pediatr Infect Dis Soc (2019) 9(2):166–72. doi: 10.1093/jpids/piy142 30715451

[143] Moore RS Kaletsky R Murphy CT. Piwi/PRG-1 Argonaute and TGF-β Mediate Transgenerational Learned Pathogenic Avoidance. Cell (2019) 177(7):1827–41.e12. doi: 10.1016/j.cell.2019.05.024 31178117 PMC7518193

[144] Christ A Günther P Lauterbach MAR Duewell P Biswas D Pelka K. Western Diet Triggers NLRP3-Dependent Innate Immune Reprogramming. Cell (2018) 172(1-2):162–75.e14. doi: 10.1016/j.cell.2017.12.013 29328911 PMC6324559

